# Temporal Effects of Surface Plasmon Polaritons in a Quantum Plasma Slab

**DOI:** 10.3390/e28050496

**Published:** 2026-04-26

**Authors:** José Tito Mendonça, José Luis Figueiredo, Hugo Terças

**Affiliations:** 1 Instituto de Plasmas e Fusão Nuclear, Instituto Superior Técnico, Universidade de Lisboa, 1049-001 Lisboa, Portugal; jose.luis.figueiredo@tecnico.ulisboa.pt; 2 Physics Department, Instituto Superior de Engenharia de Lisboa, Instituto Politécnico de Lisboa, 1959-007 Lisboa, Portugal; hugo.tercas@tecnico.ulisboa.pt

**Keywords:** plasmons, polaritons, time refraction, surface waves, quantum materials

## Abstract

The temporal effects associated with surface plasmon polaritons (SPP) in a slab of conductive quantum material (metal, graphene, or semiconductor) are described as a quantum plasma. Exchange potentials associated with quantum degeneracy are included. We derive a new dispersion relation of SPP modes in a quantum plasma slab with finite size, which reduces to the previously known cases of a single plasma boundary and of a two-dimensional slab in the appropriate limits. A new SPP instability regime due to exchange quantum effects is demonstrated. The phenomenology of time refraction and time reflection is extended to SPP, and the frequency shifts and amplitude transformations due to a time boundary are derived. Finite time boundary effects and arbitrary temporal changes of the medium are also considered.

## 1. Introduction

In recent years there has been an increasing interest in temporal optical processes, not only within conventional optics but also through novel configurations and engineered materials [1,2,3,4,5,6]. In contrast with standard spatial interfaces—where the wavevector is modified while the frequency is conserved—temporal boundaries act in a dual manner, preserving the wavevector while inducing frequency conversion. This fundamental property gives rise to a new class of wave phenomena, including time reflection and time refraction [7,8], which have no direct analogue in static media.

These ideas have been further developed within the broader framework of spacetime photonics and time-modulated media, where the optical properties of materials are varied on timescales comparable to, or shorter than, the wave period. In this regime, temporal modulation can lead to photon acceleration, parametric amplification, frequency mixing, and nonreciprocal wave propagation [9,10,11,12,13,14,15]. Such effects open new routes to overcome the fundamental limitations of conventional photonic systems, enabling broadband frequency conversion without relying on nonlinearities [9,10], dynamic control of dispersion [4,11,14], and even the realization of effective gauge fields for light [16,17,18].

From an applications perspective, time-varying media have been proposed and demonstrated in a wide range of platforms, including epsilon-near-zero materials, metasurfaces, photonic crystals, and microwave circuits [9,10,11,12,19,20]. These systems enable ultrafast optical switching, tunable spectral responses, and the generation of frequency-shifted radiation, with promising applications in signal processing, telecommunications, and quantum technologies. In particular, temporal modulation has enabled nonreciprocal devices without magnetic bias, topological frequency conversion, and time-domain analogues of spatial interferometry [15,21,22].

Despite these rapid developments, most studies have focused on bulk electromagnetic waves in effectively homogeneous media, where spatial confinement and surface effects are either negligible or treated within a classical framework. The extension of these concepts to surface-bound modes—where electromagnetic fields are strongly localized—remains comparatively unexplored. This limitation is particularly significant, as temporal modulation in such systems can couple efficiently to confined modes, potentially enhancing frequency conversion and amplification processes beyond what is achievable in bulk configurations.

Of particular relevance in this context is the rise of plasmonics as a powerful optical paradigm [23,24], together with the central role of metamaterials in spatio-temporal wave control [5,9,10,11,12]. Surface plasmon polariton (SPP) modes constitute a key element of this framework, enabling strong field confinement and subwavelength manipulation of electromagnetic radiation [25,26]. More recently, plasmonics has been extended into dynamically modulated regimes, where temporal variations of material properties lead to new functionalities such as parametric amplification and time-dependent mode conversion [20,27].

However, the role of quantum effects in surface materials is often neglected. This is particularly surprising since semiconductors, metals, and graphene-based systems—widely used in plasmonics—can operate in regimes where electrons behave as a quantum plasma even at room temperature [28,29]. In such conditions, the electron dynamics is governed not only by the classical electromagnetic response but also by Fermi pressure, quantum diffraction, and exchange-correlation effects. These contributions can significantly modify both the dispersion and the stability of collective excitations [30,31,32,33,34].

In this work, we consider the time refraction of SPP modes in a slab of conductive quantum material (metal, graphene, or semiconductor), described as a quantum plasma. Exchange potentials associated with quantum degeneracy are known to compete with thermal effects and can lead to instabilities in the electrostatic limit. Here, we demonstrate that such exchange-driven instabilities can also arise in the electromagnetic regime, thereby extending previous results on quantum surface waves and electron transport [35].

Our main focus is the emergence of time-refraction and related temporal optical phenomena in SPP propagation within a quantum plasma slab. The case of a single temporal boundary—associated with a sudden increase in plasma density—has been considered recently in the absence of quantum effects [27]. On the other hand, the dispersion of quantum SPP modes at plasma interfaces has been studied by several authors [30,31], typically under the assumption of constant density. More broadly, temporal phenomena such as time-beam splitting, temporal interference, and time-crystal formation have been explored in different contexts [21,36,37,38,39], but their connection with quantum plasmonic systems remains largely unexplored.

Here, we extend these ideas to the case of a finite quantum plasma slab, where two vacuum–plasma interfaces must be considered simultaneously. We examine in detail three fundamental properties of the temporal process: (i) the occurrence of time reflection, (ii) the frequency shift of the propagating modes, and (iii) the associated energy non-conservation and wave amplification. While these effects have been studied theoretically over the past decades and observed experimentally in various time-modulated systems—particularly in metamaterials and epsilon-near-zero platforms [19]—their manifestation in surface plasmon polaritons introduces new physical regimes, arising from the interplay between surface confinement and quantum effects. The role of exchange-driven instabilities is also analyzed.

The content of the paper is the following. In Section 2, we derive the dispersion relation of SPP modes in a slab of quantum material (metal, semiconductor or plasma). In Section 3, we determine the frequency shift of these wave modes in the presence of a sharp time-boundary. In Section 4, we derive the corresponding temporal reflection and transmission coefficients that determine the amplitude of time-refracted and time-reflected SPP modes. In Section 5, we study the case of smooth time-boundaries and establish the possible range of validity of the sharp time-boundary model. Finally, in Section 6, we state some conclusions.

## 2. SPP in a Plasma Slab

**Figure 1:**
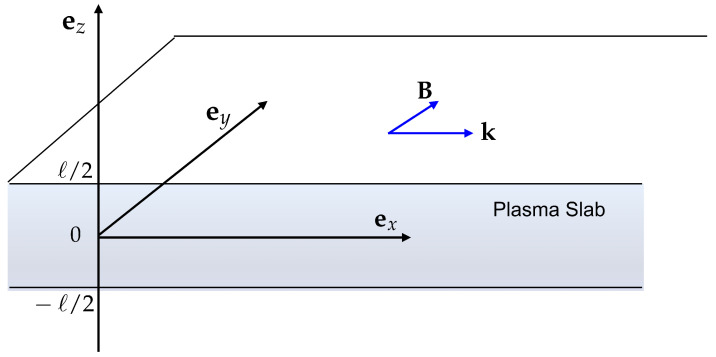
Geometric configuration: SPP modes propagation in the *y*-direction, in a plasma slab (metal or semiconductor) with size *ℓ*.

We consider a plasma slab of with size *ℓ*, such as a metal or a semiconductor, surrounded by vacuum. We use the geometry represented in Figure 1, and assume wave propagation along the *x* direction. The wave fields E and B evolve in space and time as exp(ik·r−iωt), where the wavevector is $k=ke_{x}$. In such a geometry, it is well known that the TE mode cannot propagate [23]. We therefore assume a TM mode, with magnetic field $B=B_{y}e_{y}$, and $B_{x}=B_{z}=0$. Using Maxwell’s equation, we obtain for waves oscillating at a frequency ω the following relations for the electric and magnetic field components:

(1) $$\left(\frac{\partial E_{x}}{\partial z}-ikE_{z}\right)=i\omega B_{y}\;,$$

and

(2) $$\frac{\partial B_{y}}{\partial z}=-i\frac{\omega }{c^{2}}\epsilon \left(\omega ,k\right)E_{x}\;,\;kB_{y}=-\frac{\omega }{c^{2}}\epsilon \left(\omega ,k\right)E_{z}\;,$$

where we have assumed a non-magnetic material such that $B=\mu_{0}H$. The dielectric function ϵ(ω,k) determines the displacement vector, as $D=\epsilon_{0}\epsilon \left(\omega ,k\right)E$. SI units are used in this paper. In the surrounding vacuum region we simply have ϵ(ω,k)=1. Describing the dense plasma slab as a quantum medium, we can use the following expression (see [30,31,32]):

(3) $$\epsilon \left(\omega ,k\right)=1-\frac{\omega_{p}^{2}}{\omega^{2}-u^{2}k^{2}-\hbar^{2}k^{4}/4m_{e}^{2}}\;,$$

where $\omega_{p}^{2}=e^{2}n_{0}/\epsilon_{0}m_{e}$ defines the plasma frequency. The velocity *u* can be defined as the difference between two quantum velocities, which are the Fermi velocity $v_{F}$ and the exchange velocity $v_{ex}$, according to $u^{2}=v_{F}^{2}/3-v_{ex}^{2}/2$. They also depend on the electron plasma density $n_{0}$ as

(4) $$v_{F}\equiv \frac{\hbar k_{F}}{m_{e}}={\left(3\pi^{2}\right)}^{1/3}\frac{\hbar }{m_{e}}n_{0}^{1/3}\;,\;v_{ex}\equiv \frac{\omega_{p}}{k_{F}}={\left(3\pi^{2}\right)}^{-1/3}\frac{e}{\sqrt{\epsilon_{0}m_{e}}}n_{0}^{1/6}\;.$$

Given the above definition, we realize that the quantity $u^{2}$ only depends on the plasma density $n_{0}$, and can change sign when we increase the plasma density such that the exchange term becomes dominant. This eventually leads to wave instabilities as recently discussed in the electrostatic regime [32,35]. The same can occur in the electromagnetic regime as shown below. In order to stress these properties, it is useful to write the dielectric function in normalized units to the *Fermi frequency*, $\omega_{F}\equiv E_{F}/\hbar =\hbar k_{F}^{2}/2m_{e}$. Defining the dimensionless quantities

(5) $$\Omega =\frac{\omega }{\omega_{F}}\;,\;\Omega_{p}=\frac{\omega_{p}}{\omega_{F}}\;,\;\kappa =\frac{kc}{\omega_{F}}\;,$$

and using $\nu_{F}=v_{F}/c$, we can rewrite Equation (3) as

(6) $$\epsilon \left(\Omega ,\kappa \right)=1-\frac{\Omega_{p}^{2}}{\Omega^{2}-g\left(\Omega_{p}\right)\nu_{F}^{2}\kappa^{2}-H_{F}^{2}\kappa^{4}}\;,$$

where we have used the additional quantities

(7) $$g\left(\Omega_{p}\right)=\frac{1}{3}\left(1-\frac{3}{8}\Omega_{p}^{2}\right)\;,\;H_{F}=\frac{\hbar \omega_{F}}{2m_{e}c^{2}}\;.$$

Equation (6) shows that the term in $\kappa^{4}$ is usually negligible, and that the denominator tends to zero for $\Omega /\kappa^{2}\equiv \omega^{2}/k^{2}=g\left(\Omega_{p}\right)\nu_{F}^{2}$.

It should be noticed that the main dispersion factor, evolving as $u^{2}k^{2}$, is formally identical to that of a classical plasma, where the velocity *u* is replaced by the electron thermal velocity $S_{e}=\sqrt{T_{e}/m}$. But here this factor contains two quantum contributions as defined above. One is due to the Fermi pressure that replaces the thermal pressure of classical physics. This term is, indeed, formally identical to the classical one although it relies on the Fermi velocity square and not on the thermal velocity square of a classical medium. The other term (with the opposite sign) results from the exchange potential and has no counterpart in classical physics. Finally, the dispersion factor proportional to $k^{4}$ is associated with the free streaming of quantum particles and is proportional to the ratio between the Fermi energy and the electron rest energy.

Going back to Equations (1) and (2), we can derive an evolution equation for the transverse field, from which we obtain the dispersion relation (see details in Appendix A):

(8) $$\exp\left(2\beta \ell \right)=\frac{{\left(1-\epsilon \left(\omega ,k\right)\right)}^{2}}{{\left(1+\epsilon \left(\omega ,k\right)\right)}^{2}}\;,\;\beta =\pm \sqrt{k^{2}-\frac{\omega^{2}}{c^{2}}\epsilon \left(\omega ,k\right)}\;.$$

Or, in more explicit terms

(9) $$\omega^{2}=\frac{\omega_{p}^{2}}{2}\left(1\pm e^{-\beta \ell }\right)+u^{2}k^{2}+\frac{\hbar^{2}}{4m_{e}^{2}}k^{4}\;.$$

**Figure 2:**
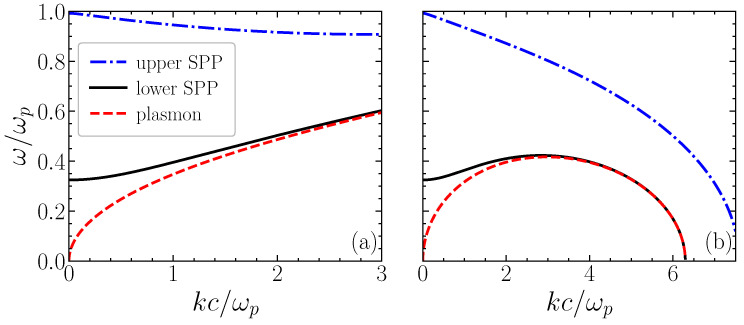
Dispersion relation of SPP modes propagation along *x*, in a plasma slab of size *ℓ*. The normalized mode frequency $\omega /\omega_{p}$ is represented as a function of the normalized wavenumber $kc/\omega_{p}$. The electromagnetic SPP dispersion (9) is represented by blue (−) and black (+) curves. The electrostatic plasmon dispersion (14) is also represented for ℓ=0.25 in wavenumber units (red curve). The quantum square velocity is assumed: (**a**) positive $\left(u^{2}/c^{2}=0.01\right)$, and (**b**) negative $\left(u^{2}/c^{2}=-0.01\right)$. The small $k^{4}$ term is ignored.

This dispersion relation is illustrated in Figure 2. In the particular case of a large slab such that βℓ≫1, this reduces to

(10) $$\omega^{2}=\frac{\omega_{p}^{2}}{2}+u^{2}k^{2}+\frac{\hbar^{2}}{4m_{e}^{2}}k^{4}\;.$$

Notice that when the exchange corrections are neglected and *u* is reduced to the Fermi velocity $v_{F}$, this coincides with the well-known expression of the dispersion relation of a surface plasmon at a single boundary [30]. In the opposite case of a very thin film, where βℓ≪1, we can identify the mode

(11) $$\omega^{2}=\frac{\omega_{p}^{2}}{2}\beta \ell +u^{2}k^{2}+\frac{\hbar^{2}}{4m_{e}^{2}}k^{4}\;.$$

In terms of the dimensionless variables (5), this expression can be rewritten as

(12) $$\Omega^{2}=\frac{\Omega_{p}}{2}\kappa \ell \left[1-\frac{\Omega^{2}}{\kappa^{2}}\epsilon \left(\Omega ,\kappa \right)\right]+g\left(\Omega_{p}\right)\nu_{F}^{2}\kappa^{2}+H_{F}^{2}\kappa^{4}\;.$$

Noting that the square bracket needs to be positive in order to satisfy a evanescent mode in the *z*-direction, we realize that the mode frequency can become negative for $g(\Omega_{p})≃0$, which means for $\Omega_{p}^{2}≃8/3$, or $v_{ex}≃v_{F}$.

Furthermore, we should notice that, in the electrostatic limit, $c^{2}\to \infty$, we can make the replacement β=k. Another useful limit corresponds to a slab with infinitesimal width ℓ→0, which reduces to the case of a two-dimensional medium. But this limit can never be attained because of the uncertainty principle, which implies that $\ell \geq \ell_{m}$, with the minimum value determined by the uncertainty of the electron momentum Δp, or

(13) $$\ell_{m}=\frac{\hbar }{\Delta p}=\frac{\hbar }{m_{e}v_{F}}=\frac{1}{k_{F}}\;,$$

This means that the dispersion relation of a surface plasmon in a two-dimensional plasma has to be given by

(14) $$\omega^{2}=\alpha k+u^{2}k^{2}+\frac{\hbar^{2}}{4m_{e}^{2}}k^{4}\;,\;\alpha =\frac{\omega_{p}^{2}}{2k_{F}}\;.$$

This expression has previously been determined by purely two-dimensional methods (see [35]). It can be seen that an instability occurs when the exchange effects dominate and $u^{2}$ becomes negative. In this case, a purely growing mode takes place for wavenumbers above a certain critical value, $k_{cr}$, with a growth rate

(15) $$\gamma \equiv ℑ\left(\omega \right)=\sqrt{|u^{2}|k^{2}-\alpha k}\;,\;k\geq k_{cr}\equiv \frac{\alpha }{|u^{2}|}\;.$$

The influence of the quartic term proportional to $k^{4}$ is usually negligible and was ignored here. Returning to Equation (11), we can see that the instability is also possible for electromagnetic modes in three-dimensions but higher values of $k\geq k_{cr}^{\prime }$ satisfying

(16) $$k_{cr}^{\prime }=\frac{\omega_{p}^{2}\ell }{2|u^{2}|}=\left(\ell k_{F}\right)k_{cr}\;.$$

Notice that the change in sign of $u^{2}$ occurs when the plasmon energy is equal to the Fermi energy $\hbar \omega_{p}≃E_{F}$. Physically, the nature of this instability can be explained in the following way. Fermi pressure (Pauli exclusion) resists density fluctuations and provides a positive restoring contribution—the quantum analogue of thermal pressure—whereas the exchange (Fock) interaction, arising from the antisymmetry of the electron wavefunction, lowers the energy of nearby electrons and introduces an effective attractive contribution to the electron–electron interactions, acting as a negative pressure. At high enough density, once $\omega_{p}\geq E_{F}$ (i.e., $\Omega_{p}\geq 8/3$), exchange dominates, $u^{2}$ changes sign and perturbations at $k>k_{cr}$ grow exponentially rather than oscillate, which is reminiscent of Jeans instability driven here by quantum correlations rather than by gravity. But this instability is not our main concern here (see [35] for a more detailed analysis).

## 3. Frequency Shifts

We now examine the occurrence of a temporal discontinuity, occurring at $t=t_{0}$, which can be described by a sudden exchange of the plasma frequency. This can be written as

(17) $$\omega_{p}^{2}\left(t\right)=\omega_{pi}^{2}\left\{1+\tilde{\epsilon }\;\Theta \left(t-t_{0}\right)\right\}\;,$$

where $\Theta (t-t_{0})$ is the Heaviside function, and $\omega_{pi}$ is the initial plasma frequency. Its final value, for $t<t_{0}$, is $\omega_{pf}=\omega_{pi}\sqrt{1+\tilde{\epsilon }}$, where $\tilde{\epsilon }=n_{f}/n_{0}-1$ determines the ratio between the initial and the final electron plasma density. This is what can be called a sharp *time-boundary*, which is similar to a space boundary of the plasma slab but exists in the temporal dimension. Notice that, at this temporal boundary, the Fermi and exchange velocities defined by Equation (4) also suffer an abrupt shift.

The validity of Maxwell’s equations at all times, including the instant $t=t_{0}$, implies time-boundary conditions that are distinct from the space-boundary conditions considered in the previous section. They now involve the fields B and D, and can be stated as

(18) $$B\left(t_{0}-\delta \right)=B\left(t_{0}+\delta \right)\;,\;D\left(t_{0}-\delta \right)=D\left(t_{0}+\delta \right)\;,$$

for δ→0. Given the invariance of the wavevector $k=ke_{x}$, the temporal boundary defined by Equation (17) implies the existence of a frequency shift associated with the same wave mode. For simplicity, we start with the electrostatic mode, valid in the quasi-two-dimensional limit. Using Equation (11) with β=k, we can see that the final frequency $\omega_{f}$ of a wave with initial frequency $\omega_{i}$ is determined by

(19) $$\omega_{f}^{2}-\omega_{i}^{2}=\frac{k\ell }{2}\left(\omega_{pf}^{2}-\omega_{pi}^{2}\right)+\left(u_{f}^{2}-u_{i}^{2}\right)k^{2}\;.$$

Or, equivalently

(20) $$\omega_{f}^{2}=\omega_{i}^{2}+\frac{\tilde{\epsilon }}{2}k\ell \omega_{pi}^{2}+\Delta u^{2}k^{2}\;,$$

where the quantity $\Delta u^{2}=\left(u_{f}^{2}-u_{i}^{2}\right)$ is a function of the initial plasma density, $n_{0i}$ and $\tilde{\epsilon }$, and can eventually change sign due to the different density dependence of the Fermi and exchange velocities, $v_{F}$ and $v_{ex}$.

If we now use the electromagnetic description of the SPP modes, satisfying the dispersion relation (9), we arrive at a new value of the frequency shift, determined by

(21) $$\omega_{f}^{2}=\omega_{i}^{2}+\frac{\tilde{\epsilon }}{2}\omega_{pi}^{2}\pm \frac{1}{2}\left(\omega_{pf}^{2}\pm e^{-\beta_{f}\ell }-\omega_{pi}^{2}\pm e^{-\beta_{i}\ell }\right)+\Delta u^{2}k^{2}\;.$$

**Figure 3:**
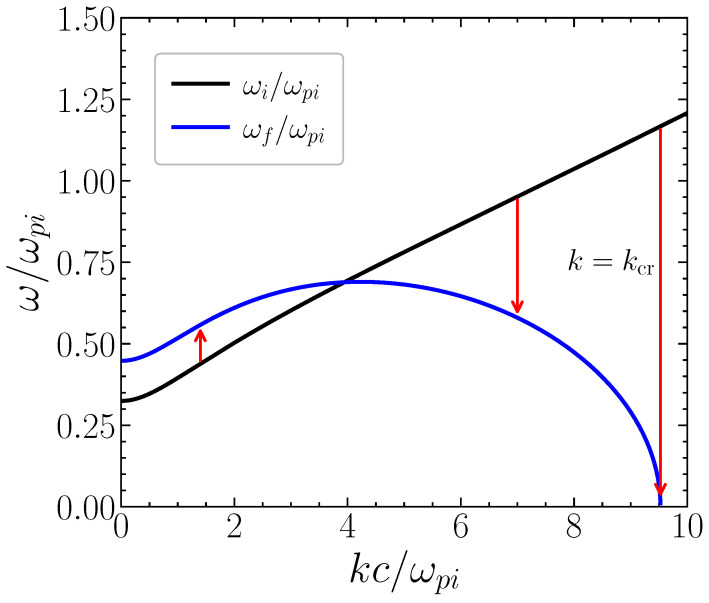
Frequency of SPP modes, before $\omega_{i}/\omega_{pi}$, and after $\omega_{f}/\omega_{pi}$ the temporal transition, as a function of the normalized wavenumber $kc/\omega_{pi}$. The possible occurrence of positive and negative frequency shifts is illustrated by the arrows in this figure. The extreme case of the wave collapse into a static SPP crystal is indicated. The black (and blue) curve corresponds to the initial (and final) SPP electromagnetic dispersion.

Here, we use the initial and final values of the parameter β, defined by

(22) $$\beta_{j}^{2}=k^{2}-\frac{\omega_{j}^{2}}{c^{2}}\left\{1-\frac{\omega_{pj}^{2}}{\omega_{j}^{2}-u_{j}^{2}k^{2}-\hbar^{2}k^{4}/4m_{e}^{2}}\right\}\;,$$

for j=(i,f). In contrast with the electrostatic case, we now have an implicit equation for the final wave frequency $\omega_{f}$. Of course, in the electrostatic limit, $c^{2}\to \infty$, we can use $\beta_{i}=\beta_{f}=k$ and recover the explicit Equation (20). These results are illustrated in Figure 3.

## 4. T and R Coefficients

The temporal boundary conditions of Equation (18) imply the creation of time-reflected signals and the eventual occurrence of wave amplification. This can be described by the so-called time-transmission and time-reflection coefficients, *T* and *R*. In order to derive their expressions, we need to consider all the field modes associated with the same wavevector k and its reverse (−k) as shown before [7,21]. The corresponding electric field can be written as

(23) $$E\left(r,t\right)=\left\{E\left(z\right)e^{-i\omega t}+E^{\prime }\left(z\right)e^{+i\omega t}\right\}e^{ikx}+c.c.\;,$$

where waves with field amplitudes E(z) and $E^{\prime }\left(z\right)$ are assumed to propagate with the same wavenumber *k* along the *x*-axis but in opposite directions.

In order to avoid ambiguities (that will be discussed at the end of this section), we consider the field continuity conditions at time $t_{0}=0$ and at specific positions $z_{\pm }=\pm \ell /2$, which correspond to the upper and lower boundaries of the plasma slab. Using the temporal continuity conditions (18), this leads to the following relations between modes moving in opposite directions:

(24) $$B_{0}\left(z_{\pm }\right)+{B^{\prime }}_{0}\left(z_{\pm }\right)=B_{f}\left(z_{\pm }\right)+{B^{\prime }}_{f}\left(z_{\pm }\right)\;,\;D_{0}\left(z_{\pm }\right)+{D^{\prime }}_{0}\left(z_{\pm }\right)=D_{f}\left(z_{\pm }\right)+{D^{\prime }}_{f}\left(z_{\pm }\right)\;,$$

where the subscripts (0,f) pertain to $\left(t=t_{0}-\delta \right)$ and $\left(t=t_{0}+\delta \right)$, respectively, for δ→0. Noting that B reduces to $B_{y}e_{y}$ and using Equation (3), we have

(25) $$B_{y}=-\frac{\omega }{kc^{2}}\epsilon \left(\omega ,k\right)E_{z}=-\frac{\omega }{\epsilon_{0}kc^{2}}D_{z}\;,$$

This allows us to write the first of Equation (24) in terms of the electric field as

(26) $$\frac{\omega_{i}}{kc^{2}}\epsilon \left(\omega_{i},k\right)\left(E_{z0}-E_{zi}^{\prime }\right)=\frac{\omega_{f}}{kc^{2}}\epsilon \left(\omega_{f},k\right)\left(E_{zf}-E_{zf}^{\prime }\right)\;.$$

Similarly, from the second of these equations, we obtain

(27) $$\epsilon \left(\omega_{i},k\right)\left(E_{zi}+E_{zi}^{\prime }\right)=\epsilon \left(\omega_{f},k\right)\left(E_{zf}+E_{zf}^{\prime }\right)\;.$$

This can be rewritten as

(28) $$\left(E_{zi}-E_{zi}^{\prime }\right)=\alpha_{z}\left(E_{zf}-E_{zf}^{\prime }\right)\;,\;\left(E_{zi}+E_{zi}^{\prime }\right)=\beta_{z}\left(E_{zf}+E_{zf}^{\prime }\right)\;,$$

with

(29) $$\alpha_{z}=\frac{\omega_{f}}{\omega_{i}}\beta_{z}\;,\;\beta_{z}=\frac{\epsilon (\omega_{f},k)}{\epsilon (\omega_{i},k)}\;.$$

These relations are valid in the plasma slab region, in close vicinity of the boundaries, $z=z_{\pm }+\epsilon$, with ϵ→0. Similar relations could also be established for the external vacuum region, where we would have $\beta_{z}=1$. We could also use the other electric field component, $E_{x}$. Using Equations (1) and (2) we can see that it is related to $E_{z}$ by

(30) $$\frac{\partial E_{x}}{\partial z}=\frac{i}{k}\beta^{2}\left(\omega \right)E_{z}\;,$$

where $\beta^{2}\left(\omega \right)$ is defined in Equation (A1). Noting that the field solutions evolve along *z* as exp[±β(ω)z], we conclude that

(31) $$\left|\frac{E_{x}}{E_{z}}\right|=\frac{1}{k}\beta \left(\omega \right)={\left(1-\frac{\omega^{2}}{k^{2}c^{2}}\epsilon \left(\omega ,k\right)\right)}^{1/2}\;.$$

This means that we can characterize the electric field amplitude $E_{z}$, and its transformation at the time-boundary $t=t_{0}$ using Equations (28) and (29). Let us then solve them for the field amplitudes $E_{zf}$ and $E_{zf}^{\prime }$. We obtain

(32) $$E_{zf}=\frac{\left(\alpha_{z}+\beta_{z}\right)}{2\alpha_{z}\beta_{z}}E_{zi}+\frac{\left(\alpha_{z}-\beta_{z}\right)}{2\alpha_{z}\beta_{z}}E_{zi}^{\prime }\;,$$

and

(33) $$E_{zf}^{\prime }=\frac{\left(\alpha_{z}-\beta_{z}\right)}{2\alpha_{z}\beta_{z}}E_{zi}+\frac{\left(\alpha_{z}+\beta_{z}\right)}{2\alpha_{z}\beta_{z}}E_{zi}^{\prime }\;,$$

These expressions show that the occurrence of a temporal discontinuity implies a change in the field amplitude of the time-transmitted wave mode, and the formation of a time-reflected signal. This is particularly obvious if we consider the case of $E_{zi}^{\prime }=0$, where initially there is no wave propagating in the negative *x*-direction. In this case, we can define transmission and reflection coefficients, *T* and *R*, such that

(34) $$T\equiv \frac{E_{zf}}{E_{zi}}=\frac{\left(\alpha_{z}+\beta_{z}\right)}{2\alpha_{z}\beta_{z}}\;,\;R\equiv \frac{E_{zf}^{\prime }}{E_{zi}}=\frac{\left(\alpha_{z}-\beta_{z}\right)}{2\alpha_{z}\beta_{z}}\;,$$

Using the explicit expressions for $\alpha_{z}$ and $\beta_{z}$ as given in (29), we get

(35) $$T\equiv \frac{E_{zf}}{E_{zi}}=\frac{1}{2}\left(1+\frac{\omega_{i}}{\omega_{f}}\right)\frac{\epsilon (\omega_{i},k)}{\epsilon (\omega_{f},k)}\;,\;R\equiv \frac{E_{zf}^{\prime }}{E_{zi}}=\frac{1}{2}\left(1-\frac{\omega_{i}}{\omega_{f}}\right)\frac{\epsilon (\omega_{i},k)}{\epsilon (\omega_{f},k)}\;.$$

**Figure 4:**
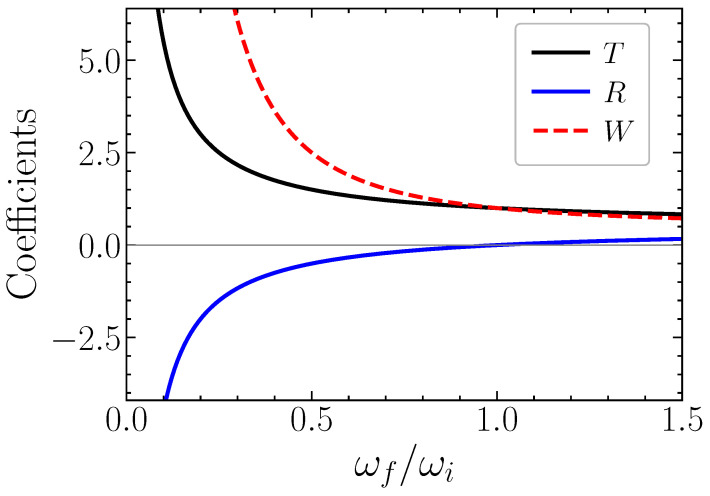
Time-transmission and -reflection coefficients, *R* and *T* for SPP modes, as a function of the mode frequency ratio $\left(\omega_{f}/\omega_{i}\right)$, as given by Equation (35). The quantity $W=R^{2}+T^{2}$, which represents the energy gain associated with time reflection and time refraction, is also shown.

In the trivial case of $\omega_{i}=\omega_{f}$ we would get T=1,R=0 as expected. This result is illustrated in Figure 4, where these coefficients are presented as a function of the frequency ratio $\left(\omega_{f}/\omega_{i}\right)$, for a sudden density perturbation. Notice that this ratio only depends on the wavenumber *k*, which remains constant. As can be seen from this figure, the energy gain $W=R^{2}+T^{2}$ due to a temporal boundary diverges for $k=k_{cr}$, when the initial wave mode can be transformed into a zero-frequency mode, such that $(\omega_{i}/\omega_{f})\to \infty$. This occurs when $\omega_{p}\to \omega_{F}$, or $\Omega_{p}\to \sqrt{3/8}$. In this case, the formation of a static *SPP crystal* is expected to occur, with an amplitude that is determined by the available electromagnetic energy in the system.

This completes our basic description of the time refraction of SPP modes in a quantum plasma slab. The derivation of the *T* and *R* coefficients was established at precise positions $z=z_{\pm }+\epsilon$, for ϵ→0. But the temporal continuity conditions imposed by Maxwell’s equations should stay valid everywhere, which means for all values of *z*. Our approach is correct in the vacuum region but leads to deviations from continuity in the plasma side. Deviations depend on the *z* position and evolve as $\exp\pm [\left(\beta_{i}-\beta_{f}\right)z]$.

But it can easily be realized that such deviations are spurious, and result from the sudden change of the medium at $t=t_{0}$. They disappear for a smooth time-boundary, where a more physically relevant boundary with a finite width is considered. For that reason, the apparent incongruence of the above method is ignored. In the next section, the case of a smooth time-boundary is briefly discussed.

## 5. Smooth Time-Boundary

Here, we consider the case where the Heaviside function appearing in Equation (17) is replaced by a hyperbolic function which provides a smooth temporal transition between the initial and final values of the electron plasma density. We therefore assume that

(36) $$\omega_{p}^{2}\left(t\right)=\omega_{pi}^{2}\left\{1+\frac{\tilde{\epsilon }}{2}\tanh\left(\nu t\right)\right\}\;,$$

where the quantity (1/ν) determines the temporal width of the transition. We return to Equations (32) and (33), and assume a time-varying dielectric function ϵ(t)≡ϵ(ω(t),k), where ω(t) is the instantaneous wave frequency. From here, we can then derive the differential evolution equations, which take the form

(37) $$\frac{\partial E_{z}}{\partial t}=\delta A\;E_{z}+\delta B\;E_{z}^{\prime }\exp\left[i\varphi \left(t\right)\right]\;,\;\frac{\partial E_{z}}{\partial t}=\delta A\;E_{z}^{\prime }+\delta B\;E_{z}\exp\left[-i\varphi \left(t\right)\right]\;,$$

where the phase is $\varphi \left(t\right)=2\int^{t}\omega \left(t^{\prime }\right)dt^{\prime }$, and the coupling coefficients are

(38) $$\delta A=-\frac{1}{2}\frac{\partial }{\partial t}\ln\left[\omega \left(t\right)\epsilon^{2}\left(t\right)\right]\;,\;\delta B=+\frac{1}{2}\frac{\partial }{\partial t}\ln\left[\omega \left(t\right)\epsilon^{-2}\left(t\right)\right]\;.$$

The coupled Equation (37) can be formally integrated, using the new field variables

(39) $$F_{z}=E_{z}\exp\left(-\delta A\right)\;,\;F_{z}^{\prime }=E_{z}^{\prime }\exp\left(-\delta A\right)\;,$$

which allows us to write

(40) $$\frac{\partial F_{z}}{\partial t}=\eta \left(t\right)\;F_{z}^{\prime }\;,\;\frac{\partial F_{z}^{\prime }}{\partial t}=\eta^{*}\left(t\right)\;F_{z}\;,\;\;\eta \left(t\right)=\delta B\;\exp\left[-i\varphi \left(t\right)\right]\;.$$

For initial field values $F_{z}\left(0\right)$ and $F_{z}^{\prime }\left(0\right)$, the solutions are

(41) $$F_{z}\left(t\right)=\cosh\left[r\left(t\right)\right]F_{z}\left(0\right)-\sinh\left[r\left(t\right)\right]F_{z}^{\prime }\left(0\right)\;,\;F_{z}^{\prime }\left(t\right)=\cosh\left[r\left(t\right)\right]F_{z}^{\prime }\left(0\right)-\sinh\left[r\left(t\right)\right]F_{z}\left(0\right)\;,$$

where we have used the *squeezing parameter*, r(t), defined as

(42) $$r\left(t\right)=\int^{t}\eta \left(t^{\prime }\right)dt^{\prime }\equiv \frac{1}{2}\int^{t}\frac{\partial }{\partial t}\ln\left[\omega \left(t^{\prime }\right)\epsilon^{-2}\left(t^{\prime }\right)\right]\exp\left[-i\varphi \left(t^{\prime }\right)\right]dt^{\prime }\;.$$

Notice that this parameter can be explicitly written in terms of the derivative of the expression defined in Equation (36). In the particular case of a single wave mode propagating initially in the positive *x*-direction, we have $F_{z}^{\prime }\left(0\right)=0$. The solutions (41) can then be reduced to the following expressions for the electric fields

(43) $$E_{z}\left(t\right)=T\left(t\right)E_{z}\left(0\right)\;,\;E_{z}^{\prime }\left(t\right)=R\left(t\right)F_{z}\left(0\right)\;,$$

where the instantaneous transmission and reflection coefficients T(t) and R(t) are determined by

(44) T(t)=cosh[r(t)]exp[−δA(t)+δA(0)],R(t)=−sinh[r(t)]exp[−δA(t)+δA(0)].

**Figure 5:**
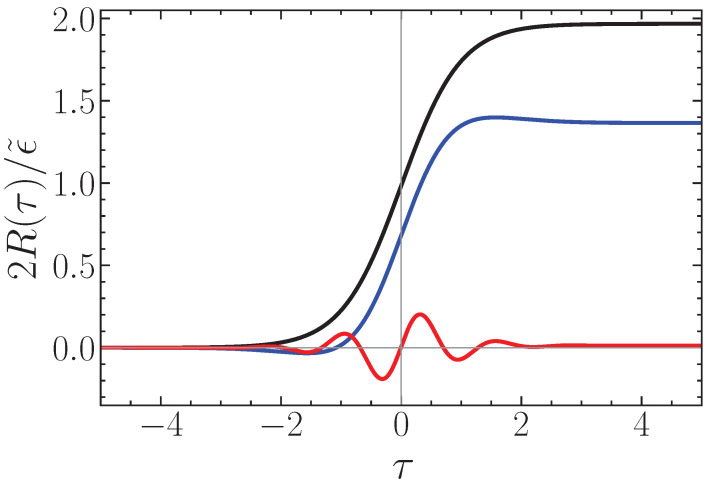
Normalized time-reflection coefficient, $2R\left(\tau \right)/\tilde{\epsilon }$ of SPP modes, as a function of normalized time τ=νt, for three distinct values of 2ω/ν=1 (black curve), 3 (blue) and 10 (red).

This completes our formal solution of the problem of a smooth temporal boundary. This solution also stays valid for the general case of an arbitrary temporal evolution of the plasma slab. See Figure 5 for three different values of the transition time scale 1/ν. We can see that the formation of a reflected wave is stronger when ν≃2ω, and nearly vanishes for slow time changes, when ν/2ω≪1. The simple sharp time-boundary model explored in Section 3 and Section 4 is valid when the temporal transition is faster than the period of the SPP mode, or ν≥2ω.

## 6. Conclusions

We have studied the problem of time refraction for surface waves that can be excited in a quantum plasma slab. This problem involves field continuity conditions in space and in time, which are not identical to each other. Both electromagnetic and electrostatic wave modes were considered. The case of slabs with an infinitesimal width is reduced to a quasi two-dimensional problem, and was also studied in detail. We have derived the wave frequency shifts and the time-transmission and time-reflection coefficients, which are similar to those valid in spatially homogeneous media. The general case of smooth temporal transitions and arbitrary temporal variations of the slab density was formally solved.

It should be stressed that several new results concerning SPP modes, associated with both stationary and time-varying plasma slabs, have been discussed here. First, we derived a new dispersion relation of SPP modes in a quantum plasma slab with finite size *ℓ* where exchange effects were included as stated in Equations (8) and (9). In the appropriate limits of ℓ→∞ and ℓ→0, they reduce to the previously known results of a single plasma boundary and of a two-dimensional slab as it should be expected.

Second, a new instability of surface plasmon polaritons due to exchange quantum effects was shown to exist, with growth rate given by Equation (15). This extends the exchange instability recently derived for electrostatic modes in 2D [35], in order to include electromagnetic surface waves and finite size effects.

Third, the phenomenology of time refraction and time reflection was extended to surface plasmon polaritons, and the frequency shifts of these modes due to the occurrence of a time boundary were derived. They are given by Equations (21) and (22). The corresponding wave amplitude transformations were established as given by Equations (34) and (35). Finally, the demonstration that a finite time transition only creates a time reflected signal if its duration is of the order or shorter than the wave period is stated by Equation (44) and illustrated in Figure 5.

Extension of this work to the case of temporal beam splitters [21] and time crystals [36] is straightforward, and can be derived from the present results, for sharp as well as for smooth temporal boundaries. Another possible extension is related with superluminal fronts, using electron-ion plasmas [37,38] or metamaterials [3,25], which can be related to the present work by a simple Lorentz transformation [39]. Some of these scenarios will be examined in a separate work.

In our present analysis, we have neglected wave losses and assumed that the collision frequency is much smaller than the wave frequency. These losses can be due to particle collisions, scattering of the electromagnetic modes and Landau damping of the electrostatic ones. They can easily be integrated in the above description if necessary.

Finally, it should be noticed that plasmonics is a very active field, where the present ideas could be explored in many different directions. As examples of the possible application of temporal effects, we should mention topological plasmons [40,41], plasmonic induced transparency [42,43], or bound states in the continuum [44,45]. A vast area of research is therefore open for exploration.

## Data Availability

The data used in the paper is contained in the figures.

## Appendix A. Derivation of Dispersion Relation

Starting from Equations (1) and (2) we derive an equation for the transverse field, of the form

(A1) $$\frac{\partial^{2}B_{y}}{\partial z^{2}}-\beta^{2}\left(\omega ,k\right)B_{y}=0\;,\;\beta^{2}\left(\omega ,k\right)=k^{2}-\frac{\omega^{2}}{c^{2}}\epsilon \left(\omega ,k\right)\;.$$

For $\beta^{2}>0$, this leads to the following field solutions, evanescent along the *z*-direction. In the vacuum region, we should have $\beta_{0}^{2}\equiv k^{2}-\omega^{2}/c^{2}>0$. The field solutions in the upper-vacuum region (z>ℓ/2) are then given by

(A2) $$B_{y}\left(z\right)=A\exp\left(-\beta_{0}z\right)\;\;E\left(z\right)=A\frac{c^{2}}{\omega }\left(i\beta e_{x}-ke_{z}\right)\exp\left(-\beta_{0}z\right)\;,$$

where the constant *A* is an amplitude. In the lower-vacuum region (z<−ℓ/2) this is replaced by

(A3) $$B_{y}\left(z\right)=B\exp\left(+\beta_{0}z\right)\;\;E\left(z\right)=-B\frac{c^{2}}{\omega }\left(i\beta e_{x}+ke_{z}\right)\exp\left(+\beta_{0}z\right)\;,$$

where *B* is another amplitude. Finally, in the intermediate region (−ℓ/2<z<ℓ/2), the evanescent field solutions become

(A4) $$B_{y}\left(z\right)=C\exp\left(i\beta z\right)+C^{\prime }\exp\left(-i\beta z\right)\;,$$

and

(A5) $$E\left(z\right)=\frac{c^{2}}{\omega \epsilon (\omega ,k)}\left(i\beta e_{x}-ke_{z}\right)\left\{-C\exp\left(+\beta z\right)+C^{\prime }\exp\left(+\beta z\right)\right\}\;,$$

with new amplitudes *C* and $C^{\prime }$. The continuity conditions between the fields $B_{y}$ and $E_{x}$ at the upper boundary (z=ℓ/2) imply the following relations between the different amplitudes:

(A6) $$A\;e^{-\beta_{0}\ell /2}=C\;e^{\beta \ell /2}+C^{\prime }\;e^{-\beta \ell /2}\;,\;A\;e^{-\beta_{0}\ell /2}=-\frac{1}{\epsilon (\omega ,k)}\left\{C\;e^{\beta \ell /2}+C^{\prime }\;e^{-\beta \ell /2}\right\}\;.$$

Similar relations can be established at the lower boundary (z=−ℓ/2), and lead to

(A7) $$B\;e^{-\beta_{0}\ell /2}=C\;e^{-\beta \ell /2}+C^{\prime }\;e^{\beta \ell /2}\;,\;B\;e^{-\beta_{0}\ell /2}=\frac{1}{\epsilon (\omega ,k)}\left\{C\;e^{\beta \ell /2}-C^{\prime }\;e^{\beta \ell /2}\right\}\;.$$

This implies a double equality relation between the internal field amplitudes *C* and *D*, of the form

(A8) $$C\;e^{\beta \ell }+C^{\prime }=-\frac{C}{\epsilon (\omega ,k)}e^{\beta \ell }+\frac{C^{\prime }}{\epsilon (\omega ,k)}\;,\;C+C^{\prime }\;e^{\beta \ell }=-\frac{C}{\epsilon (\omega ,k)}-\frac{C^{\prime }}{\epsilon (\omega ,k)}e^{\beta \ell }\;,$$

This can also be written as

(A9) $$C^{\prime }\left(1-\epsilon \left(\omega ,k\right)\right)=Ce^{\beta \ell }\left(1+\epsilon \left(\omega ,k\right)\right)\;,\;C\left(1-\epsilon \left(\omega ,k\right)\right)=C^{\prime }e^{\beta \ell }\left(1+\epsilon \left(\omega ,k\right)\right)\;.$$

From here we obtain the dispersion relation (8).
